# Occurrence of diverse circoviruses in wild birds in Hungary

**DOI:** 10.1186/s13567-025-01696-5

**Published:** 2026-01-09

**Authors:** Anna Pataki, Dóra Máté, Eszter Kaszab, Krisztina Bali, Krisztina Ursu, Enikő Fehér

**Affiliations:** 1 Department of Microbiology and Infectious Diseases, University of Veterinary Medicine Budapest, Hungária krt. 23-25, Budapest, 1143 Hungary; 2 National Laboratory for Infectious Animal Diseases, Antimicrobial Resistance, Veterinary Public Health and Food Chain Safety, István utca 2, Budapest, 1078 Hungary; 3 Department of Bioinformatics, One Health Institute, Faculty of Health Sciences, University of Debrecen, Nagyerdei krt. 98, Debrecen, 4032 Hungary; 4 Vetcontrol Ltd, Déli-bekötő út 8, Budapest, 1211 Hungary; 5 National Laboratory of Virology, Szentágothai Research Centre, University of Pécs, Ifjúság útja 20, Pécs, 7624 Hungary

**Keywords:** CRESS DNA virus, circovirus, cyclovirus, avian, pathogen

## Abstract

Circoviruses (CVs) infect a wide range of avian hosts, including domestic, ornamental and wild birds. The immunosuppressive effect of these viruses could make the host more susceptible to other pathogens. This study aimed to assess the potential hosts and genetic features of CVs or other related viruses in wild birds. Cloacal swab samples of 588 birds were processed for screening of circoviral sequences using nested PCR. Altogether, 19 complete genome and 21 partial sequences of small, circular, rep-encoding DNA viruses were determined, 32 of which belonged to CVs. Along with some newly established CV species (virus name long-eared owl-associated CV1 and barn owl-associated CV1), genomic sequences of previously characterized avian CVs (pigeon CV, duck CV, goose CV, gull CV, swan CV and little bittern CV) were identified. Pathogenic CVs and CVs of unknown aetiology occur in wild birds taxonomically distant from the originally described host species, such as the duck CV and pigeon CV in white stork (*Ciconia ciconia*), as well as pigeon CV in peregrine falcon (*Falco peregrinus*) and black-headed gull (*Chroicocephalus ridibundus*). The results draw attention to the widespread distribution of these viruses among wild birds which, by hiding in the host and reducing defensibility, could pose a threat to both poultry farming and efforts to wild bird conservation.

**Supplementary Information:**

The online version contains supplementary material available at 10.1186/s13567-025-01696-5.

## Introduction

In relation to poultry production, a fundamental concern is that wild birds pose a threat to farmed animals. Indeed, wild birds can transmit some pathogens, such as influenza and Newcastle disease virus, through droppings, respiratory droplets and plumage, contaminating the feed, water and environment [1–4]. If spillover occurs from wild birds into domestic flocks, in cases where domestic birds are kept in high density, pathogens can rapidly spread. Disease outbreaks can lead to economic losses through slow growth rates, reduced egg production and increased mortality in poultry flocks [1, 3] However, attention must be also paid to the processes by which potential pathogens are released into the wider environment from domestic to wild animals [1, 2]. Even if a pathogen does not actively replicate within a wild bird, the animal may serve as a transmitter. The pathogen may spillover to novel hosts through shared resources and spread quickly by long-distance avian movements [1–3]. Agricultural intensification, the sacrifice of natural habitats for meat and feed production, makes wild bird populations increasingly vulnerable [1, 2].

Circoviruses (CVs; *Circovirus* genus, *Circoviridae* family, *Cressdnaviricota* phylum) possess small, circular, single-stranded DNA genomes of ~1600–2200 bases in length, with an ambisense arrangement in respect of the open reading frames of the replication-associated (Rep) and capsid (Cp) proteins [5, 6]. Cycloviruses (CyVs), members of the *Cyclovirus* genus of the *Circoviridae* family, have similar genomic properties, but the *rep* is located on the complementary strand compared to that of CVs [5–7]. CVs and CyVs have been detected in a diverse range of hosts, including mammals, birds, fish and insects, as well as in environmental samples such as water and soil [5–22]. Several other related small, circular, Rep-encoding single-stranded (CRESS) DNA viruses have been identified; however, data remain very limited regarding their host range, geographic distribution, genetic variability and tissue tropism. This is complicated by the fact that vast majority of these viruses cannot be propagated [23, 24].

Of the viral groups mentioned here, the pathogenic role of some CVs has been confirmed. Porcine CV2 and porcine CV3, beak and feather disease virus (BFDV), pigeon CV (PiCV), goose CV (GoCV), duck CV (DuCV), finch CV and canary CV have been associated with severe diseases in their hosts [25–35]. In general, CV infection of birds may lead to lethargy, weight loss, feathering disorders, beak deformities, diarrhoea and internal organ failure [27–31, 33, 34]. CV can induce immunosuppression; thus, the host may become susceptible for secondary infections and opportunistic pathogens, which can cause more severe forms of illnesses [27–31, 33, 34].

The aim of the study was to survey CVs and their potential host range among wild birds, as well as genetic characterization of the identified viruses. In total, complete genomes of 16 CVs and 3 other CRESS DNA viruses were determined, together with 21 partial sequences. A diverse picture in terms of host species was also obtained, confirming that some CVs may be widespread in the wild.

## Materials and methods

### Sample preparation and PCR

In total, 588 cloacal swab samples, collected for influenza virus surveillance throughout Hungary, were provided by the Veterinary Diagnostic Directorate, National Food Chain Safety Office, Budapest, Hungary. Sampled wild birds and sites of collection are listed in Additional file 1, while Additional file 2 includes the sites of collection of avian samples in which CRESS DNA virus sequences were identified. The nucleic acid was extracted with the KingFisher Flex system (ThermoFisher, Waltham, Ma, USA) using the MagAttract 96 cador Pathogen Kit (Qiagen, Hilden, Germany) according to the manual.

Broad-spectrum nested PCR was used for CV detection described by Li et al. [7] with the primers CV-F1 (5′-GGIAYICCICAYYTICARGG) and CV-R1 (5′-AWCCAICCRTARAARTCRTC) in the first PCR, and with CV-F2 (5′-GGIAYICCICAYYTICARGGITT) and CV-R2 (5′-TGYTGYTCRTAICCRTCCCACCA) for the second PCR [7]. The PCR mixtures contained 1× DreamTaq Green buffer, 250 μM of dNTP mix, 250 nM of primers, 0.5 U of DreamTaq DNA Polymerase (Thermo Fisher Scientific, Waltham, MA, USA) and 1 μl of nucleic acid templates in 20 μl final volume. The cycling protocol of the PCR was as follows: denaturation at 95 °C for 3 min; 40 cycles of denaturation at 95 °C for 30 s, 52 °C or 56 °C annealing (for the first and second PCR, respectively) for 30 s, and elongation at 72 °C for 1 min; final extension at 72 °C for 10 min. PCR products of ~400 bp in length were purified from agarose gel with Geneaid Gel/PCR DNA Fragments Extraction Kit (Geneaid Biotech, Taipei, Taiwan) and were directly sequenced with the CV-F2 and CV-R2 primers.

Inverse PCR primers were designed based on the results of the direct sequencing and were used in variable combinations for the amplification of the undetermined genomic regions of the respective virus (Additional file 3). If multiple products were generated for a sample, they were submitted for next-generation sequencing as a mixture. The PCR mixture contained 1× Phusion Green HF buffer, 200 μM of dNTP mix, 200 nM of primers, 3% DMSO, 0.25 U of Phusion DNA Polymerase (Thermo Fisher Scientific, Waltham, MA, USA) and 2 μl of the extracted nucleic acid in 25 μL final volume. The cycling protocol of PCRs was as follows: denaturation at 98 °C for 30 s; 45 cycles of denaturation at 98 °C for 10 s, annealing for 30 s at a temperature calculated for each primer, and elongation at 72 °C for 2 min; final extension at 72 °C for 5 min. The PCR products were purified from agarose gel with Geneaid Gel/PCR DNA Fragments Extraction Kit (Geneaid Biotech, Taipei, Taiwan) and were prepared for next-generation sequencing.

### Next-generation sequencing

DNA libraries were prepared with the Illumina Nextera XT DNA Library Preparation Kit (Illumina, San Diego, CA, USA) combined with the Nextera XT Index Kit v2 Set C (Illumina, San Diego, CA, USA) [18]. Libraries were pooled and loaded into iSeq 100 i1 reagent cartridge v2, and were sequenced using the Illumina iSeq100 platform (Illumina, San Diego, CA, USA).

### Bioinformatic analysis

The quality of raw reads was checked with the FastQC tool [36]. Based on the results, ten bases were trimmed from both the 3′ and 5′ ends of the reads. *De novo* assembly of the sequence reads occurred with the Tadpole assembler of the Geneious Prime software v.2025.1.2 (Biomatters Ltd., Auckland, New Zealand), and the contigs were identified with the Basic Local Alignment Search Tool (BLAST). Mapping to the reference (Geneious assembler and default parameters) was performed using the closest references found based on BLAST analysis (Additional file 4). Raw reads were remapped to the assembled genomes for another checking and the final contigs were merged with the sequences obtained by nested PCR and Sanger sequencing. Open reading frames were predicted with the Open Reading Frame Finder online software and were checked through BLAST analysis. Sequences were edited with Aliview and were aligned with the MAFFT algorithm implemented in Geneious Prime software [37]. Phylogenetic analyses were carried out with the PhyML 3.0 online tool, the best calculated models and SH-like branch support [38]. The phylogenetic trees were visualized and edited with the MEGA X software [39]. Pairwise nucleotide (nt) and amino acid (aa) identities were calculated and compared with Geneious Prime and SDTv1.2 software according to Varsani et al. using CV reference sequences found by BLAST search (Table 1) [40, 41]. The pairwise identities generated by the SDTv1.2 were imported into the RStudio environment (R version 4.5.1; RStudio version 2025.5.1.513) [42, 43]. The visualization of the data in a raincloud plot was performed using the packages ggplot2 package (v3.5.2), gghalves package (v0.1.4) and tidyverse package (v2.0.0) [44–46].

**Table 1:** **CRESS DNA viruses detected in the sampled wild birds**

Virus name	Virus strain	GenBank accession no	Sampled bird	Rep aa identity with references	Highest nt identity with reference
*Duck circovirus*	*DuCV-Hun1*	*PV972690*	*White stork*	*100% with EF451157*	*99.7% with PV649664*
Duck circovirus	DuCV-Hun2	PX097028	White stork	100% with PV649664	99.8% with PV649664
*Pigeon circovirus*	*PiCV-Hun1, n* = *2*	*PV972689*	*Peregrine falcon*	*98.1% with PV454393*	*97.7% with KF738870*
Pigeon circovirus	PiCV-Hun2	PX097029	White stork	99.2% with KX108786	99.2% with OR801818
Pigeon circovirus	PiCV-Hun3	PX097030	White stork	99.2% with OR999265	98.9% with OR999239
Pigeon circovirus	PiCV-Hun4	PX097031	Black-headed gull	100% with MW181952	97.8% with DQ915950
Goose circovirus	GoCV-Hun4	PX097032	Greylag goose	100% with OK070803	99.8% with OK070803
Goose circovirus	GoCV-Hun5	PX097033	Greylag goose	98.5% with OK070803	99.3% with OK070803
Goose circovirus	GoCV-Hun6	PX097034	Greylag goose	95.0% with KT808657	92.9% with KR869727
Goose circovirus	GoCV-Hun7	PX097035	Greylag goose	100% with OK070803	99.8% with OK070803
*Swan circovirus*	*SwCV-Hun1, n* = *6*	*PV972694*	*Mute swan*	*98.6% with EU056309*	*98.5% with EU056309*
Swan circovirus	SwCV-Hun2	PX097036	Mute swan	98.5% with EU056309	97.1% with EU056309
*Gull circovirus*	*GuCV-Hun1*	*PV972695*	*Black-headed gull*	*98.3% with JQ685854*	*98.9% with JQ685854*
*Little bittern circovirus*	*TorCV1-Hun2*	*PV972691*	*Great egret*	*100% with MZ710934*	*99.4% with MZ710934*
*Little bittern circovirus*	*TorCV1-Hun3*	*PV972692*	*Great egret*	*100% with MZ710934*	*99.6% with MZ710934*
*Little bittern circovirus*	*TorCV1-Hun4*	*PV972693*	*Great egret*	*100% with MZ710934*	*99.6% with MZ710934*
Little bittern circovirus	TorCV1-Hun5	PX097037	Great egret	100% with MZ710934	100% with MZ710934
Little bittern circovirus	TorCV1-Hun6	PX097038	Great egret	99.24% with MZ710934	99.7% with MZ710934
Little bittern circovirus	TorCV1-Hun7	PX097039	Great egret	100% with MZ710934	99.7% with MZ710934
Little bittern circovirus	TorCV1-Hun8	PX097040	Spoonbill	100% with MZ710934	100% with MZ710934
*Long-eared owl-associated circovirus 1*	*le-owlCV1-Hun1*	*PV972686*	*Long-eared owl*	*70.2% with KF385421*	*57.7% with KF385421*
*Barn owl-associated circovirus 1*	*barn-owlCV1-Hun1*	*PV972687*	*Barn owl*	*88.5% with NC_055153*	*77.9% with NC_055153*
*Barn owl-associated circovirus 1*	*barn-owlCV1-Hun2*	*PV972688*	*Barn owl*	*87.8% with NC_055153*	*77.5% with NC_055153*
Barn owl-associated circovirus 1	barn-owlCV1-Hun3	PX097041	Barn owl	92.1% with NC_055153	85.4% with NC_055153
Barn owl-associated circovirus 1	barn-owlCV1-Hun4	PX097042	Barn owl	89.9% with NC_055153	85.1% with NC_055153
Swan-associated circovirus 1	SwACV1-Hun1	PX097043	Mute swan	55.6% with KR869727	64.7% with KR869727
Stork-associated cyclovirus 1	StorkACyV1-Hun1	PX097044	White stork	73% with NC_038393	74.2% with NC_038393
Bat faeces-associated cyclovirus 2	BatACyV2-Hun1	PX097045	Long-eared owl	97.2% with KY302865	94.6% with KY302865
*Ciconia ciconia-associated CRESS DNA virus 1*	*Cic-cic-CRESS1-Hun1*	*PV972697*	*White stork*	*66.6% with MZ556143*	*58% with MZ556143*
*Ciconia ciconia-associated CRESS DNA virus 2*	*Cic-cic-CRESS2-Hun1*	*PV972698*	*White stork*	*43.3% with MT206491*	*46.3% with MT206491*
Ciconia ciconia-associated CRESS DNA virus 3	Cic-cic-CRESS3-Hun1	PX097046	White stork	30% with MK947371	40% with MK947371
Ciconia ciconia-associated CRESS DNA virus 4	Cic-cic-CRESS4-Hun1	PX097047	White stork	56.5% with OM154274	56.5% with OM154274
*Platalea leucorodia-associated CRESS DNA virus*	*Plat-leu-CRESS-Hun1*	*PV972696*	*Spoonbill*	*39.8% with OQ599924*	*71.7% with HQ638064*
*Ardea cinerea*-associated CRESS DNA virus 1	Ar-Ci-CRESS1-Hun1	PX097048	Grey heron	75.4% with MW182835	81% with MW182835

The table represents the highest pairwise identity values to the closest GenBank reference sequences identified by BLAST search. The highest nucleotide identity values show genome-wide identities for complete genome sequences (italics), and identity of the replication-associated protein coding gene in case of partial sequences (normal lettering). Two pigeon circovirus, as well as six swan circovirus complete genome sequences were identical and were detected in samples of the same avian species for the respective virus, thus were assigned with one GenBank accession number (PV972689 for the pigeon circovirus and PV972694 for the swan circovirus). aa, amino acid; nt, nucleotide; Rep, replication-associated protein.

## Results

**Figure 1:**
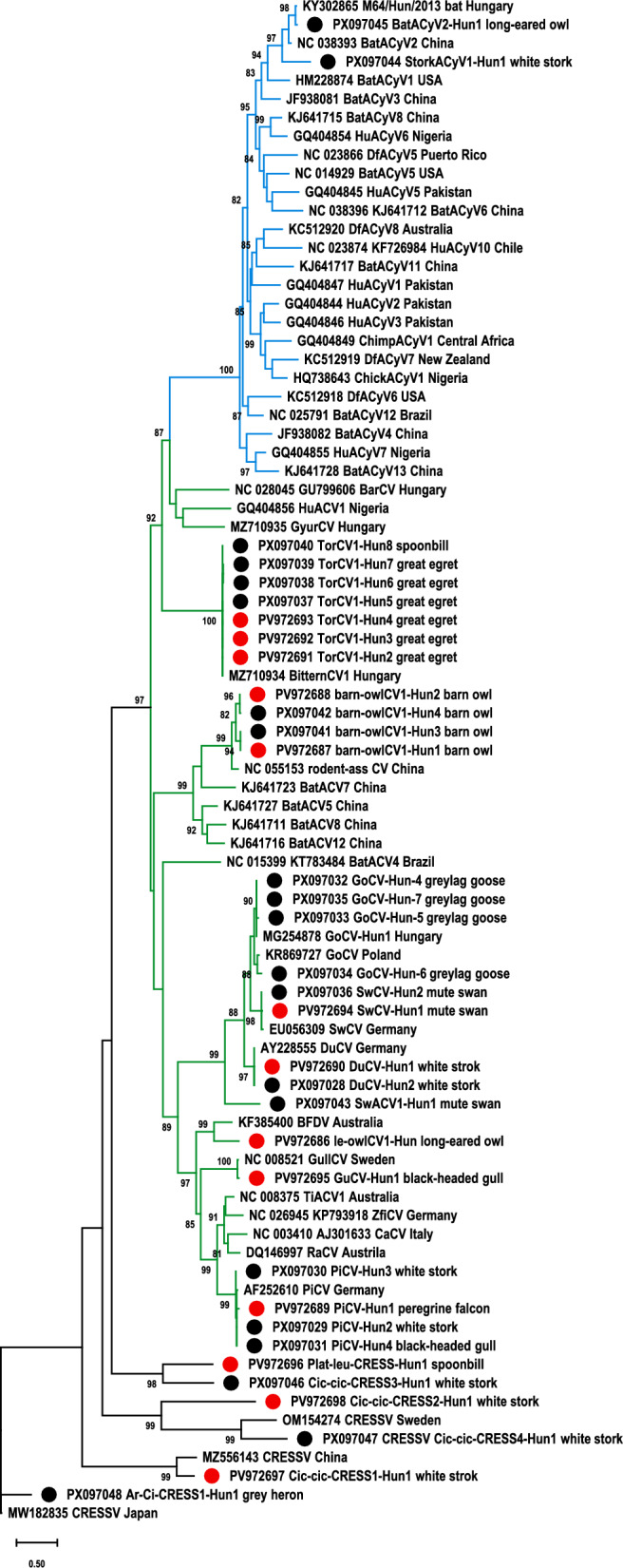
**Unrooted, maximum likelihood phylogenetic tree of CRESS DNA virus replication-associated protein (Rep) amino acid sequences, generated with the PhyML software, Q.pfam + R + F model, SH-like branch support**. Branch support values <80 were hidden. The scale bar indicates substitutions per site. Circoviruses and cycloviruses are highlighted with green and blue branch colours, respectively. Partial Rep sequences are labelled with black circles, while red circles refer to viruses where complete genome sequences were determined.

Sanger sequencing confirmed amplification of CRESS DNA virus *rep* sequences in 40 of the 588 (6.8%) cloacal swab samples. According to BLAST and phylogenetic analysis of the partial Rep, 32 aa sequences clustered with CVs and two with CyVs, while six were distantly related to unclassified CRESS DNA viruses (Table 1, Figure 1).

**Figure 2:**
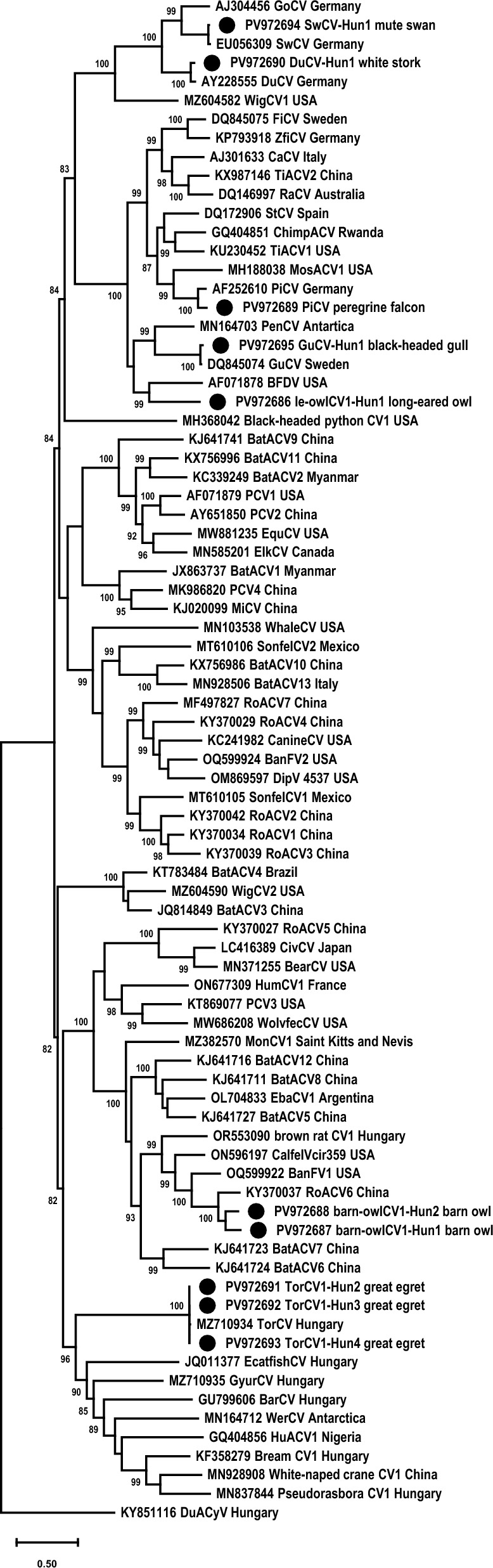
**Maximum likelihood phylogenetic tree of complete genome sequence of circoviruses rooted to the reverse complement of the duck-associated cyclovirus genome sequence**. The tree was generated with the PhyML software, GTR + R model, SH-like branch support. Branch support values <80 were hidden. The scale bar indicates substitutions per site. The novel strains were labelled with black circles.

Complete genome sequence of 19 viruses was determined combining sequences of the diagnostic and inverse PCR products. The references, used for read mapping, and statistical data (total and assembled read number, mean coverage) of the next-generation sequencing are listed in Additional file 4. Based on the structure of the complete genome, sequence comparisons and phylogeny, 16 of the viruses were found to belong to the *Circovirus* genus, while three were unclassified CRESS DNA viruses (Figure 2, Table 2) [41].

**Table 2:** **Location of the predicted open reading frames in the genome of circoviruses and unclassified CRESS DNA viruses identified in the present study**

	Genome length	*rep*	*cp*	5′ IR/IR upstream rep	3′ IR/IR downstream rep
Long-eared owl-associated circovirus 1, PV972686	2011 nt	nt 120–998 (292 aa)	nt 2002–1181 (274 aa)	nt 2003–119 (228 nt)	nt 999–1180 (182 nt)
Barn owl-associated circovirus 1, PV972687	1688 nt	nt 140–1051 (303 aa)	nt 1665–1048 (205 aa)	nt 1666–139 (162 nt)	-
Barn owl-associated circovirus 1, PV972688	1695 nt	nt 147–1058 (303 aa)	nt 1–1 and 1695–1055 (213 aa)	nt 2–146 (145 nt)	-
Pigeon circovirus (*n* = 2), PV972689	2042 nt	nt 48–1001 (317 aa)	nt 1996–1172 (274 aa)	nt 1997–47 (93 nt)	nt 1002–1171 (170 nt)
Duck circovirus, PV972690	1949 nt	nt 55–933 (292 aa)	nt 1893–1120 (257 aa)	nt 1894–54 (110 nt)	nt 934–1119 (186 nt)
Little bittern circovirus, PV972691- PV972693	1935 nt	nt 52–999 (315 aa)	nt 1858–1229 (209 aa)	nt 1859–51 (128 nt)	nt 1000–1228 (nt 229)
Swan circovirus (*n* = 6), PV972694	1783 nt	nt 55–936 (293 aa)	nt 1730–975 (251 aa)	nt 1731–54 (107 nt)	nt 937–974 (38 nt)
Gull circovirus, PV972695	2035 nt	nt 107–1024 (305 aa)	nt 1934–1197 (245 aa)	nt 1935–106 (207 nt)	nt 1025–1196 (172 nt)
Ciconia ciconia-associated CRESS DNA virus 1, PV972697	2313 nt	nt 39–1112 (357 aa)	nt 1193–1945 (250 aa)	nt 1946–38 (406 nt)	nt 1113–1192 (80 nt)
Ciconia ciconia-associated CRESS DNA virus 2, PV972698	1818 nt	nt 181–1128 (315 aa)	nt 1762–1262 (166 aa)	nt 1763–180 (236 nt)	nt 1129–1261 (133 nt)
Platalea leucorodia-associated CRESS DNA virus, PV972696	2298 nt	nt 79–978 (299 aa)	nt 2150–1059 (363 aa)	nt 2151–78 (226 nt)	nt 979–1058 (80 nt)

The first nucleotide of the predicted nonanucleotide motif was considered as nt 1 of the genome. aa, amino acid; *cp*, capsid protein coding gene; nt, nucleotide; *rep*, replication-associated protein coding gene.

**Figure 3:**
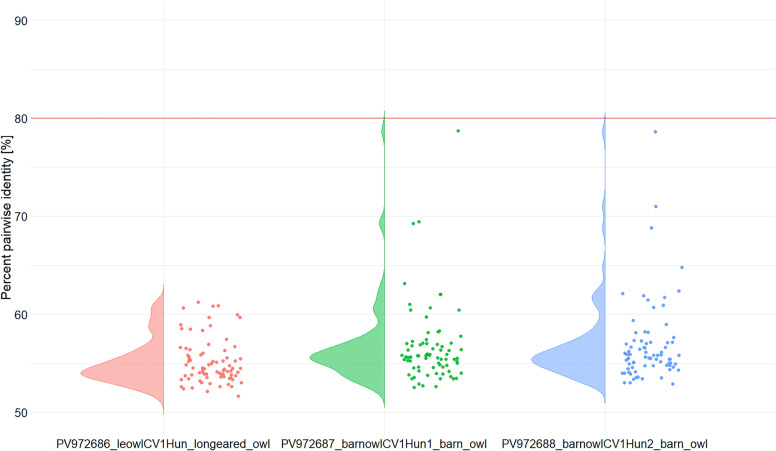
**Genome-wide pairwise identity values calculated for the novel circovirus sequences (long-eared owl-associated circovirus 1, strain le-owlCV1-Hun1, GenBank accession number PV972686 in red; barn owl-associated circovirus 1, strain barn-owlCV1-Hun1, GenBank accession number PV972687 in green; barn owl-associated circovirus 1, strain barn-owlCV1-Hun2, GenBank accession number PV972688 in blue) using reference sequences of all circoviruses (all circovirus species) and applying the matrix generated by SDTv1.2 software.**

**Figure 4:**
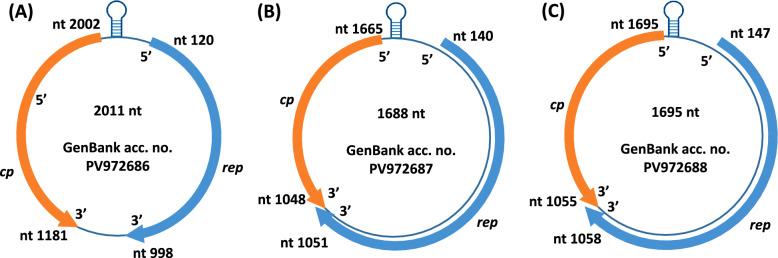
**Schematic representation of the genomic structure of the long-eared owl-associated circovirus 1 (A), and the two strains of the barn owl-associated circovirus 1 (B and C).**

The PiCV (*n* = 2, from peregrine falcon, *Falco peregrinus*), DuCV (*n* = 1, from white stork, *Ciconia ciconia*), little bittern CV (TorCV; *n* = 3, from great egret, *Ardea alba*), swan CV (SwCV; *n* = 6, from mute swan, *Cygnus olor*) and gull CV (GuCV; *n* = 1, from black-headed gull, *Chroicocephalus ridibundus*) complete genome sequences represented high pairwise nt identities with reference sequences (Table 1, Figures 1 and 2). The PiCV and SwCV complete genome sequences obtained from this study were identical among each other, and originated from specimens of the same bird species, thus, these were referred with one accession number for the respective virus (PV972689 for the PiCV and PV972694 for the SwCV sequences). Three viruses formed two separate branches among CVs in the phylogenetic tree (Figure 2). The genomic sequence of the three strains (GenBank accession nos. PV972686, PV972687 and PV972688) showed ≤80% genome-wide pairwise identity with CV reference sequences of all species of the *Circovirus* genus (Table 1, Figure 3). In addition, the genomic structure of the here-characterized viruses is consistent with that of described for other CVs (Figure 4). Thus, these could be considered as members of new CV species [41]. One of the viruses, distantly related to BFDV, originated from long-eared owl (*Asio otus*) and was named long-eared owl-associated circovirus 1 (tentative species name *Circovirus fulesbagoly*, FulCV; GenBank accession no. PV972686) (Figure 2). The other two strains were grouped with the rodent-associated circovirus 6 and were listed as barn owl-associated circovirus 1 (tentative species name *Circovirus gyongybagoly*, GyoCV; GenBank accession nos. PV972687 and PV972688) after the sampled birds (barn owl, *Tyto alba*) (Figure 2). The two GyoCV strains showed 83.4% genome-wide identity with each other and had comparable genomic structure (Figure 4). The nonanucleotide motif upstream the *rep*, and Rep aa motifs of the novel CVs (RCR and Walker motif, motif C, Arg motif), as well as the arginine-rich N terminus of the Cp were characteristic of CVs (Table 3) [41]. The predicted *cp* of the FulCV, similarly to certain avian CVs (such as BFDV), started with alternative start codon [41].

**Table 3:** **The nonanucleotide motif, as well as amino acid motifs of the replication-associated protein identified in the novel circovirus genomes**

	Long-eared owl-associated circovirus 1, PV972686	Barn owl-associated circovirus 1, PV972687 and PV972688
Nonanucleotide motif	TAGTATTAC	TAGTATTAC
RCR motif I	CFTLNN	CFTVNN
RCR motif II	PHLQG	PHLQG
RCR motif III	DNLKYCSK	QNQTYCQK
Walker-A motif	GPPGCGKSRWAS	GPPGVGKTRYAV
Walker-B motif	VMDDY	MLDDF
Motif C	ITSN	ITSN
Arg finger motif	ALFRRF	AMFRRI

Regarding the partial Rep aa sequences, 16 clustered with CVs and 2 with CyVs, while 3 with unclassified CRESS DNA virus sequences (Figure 1). Four of the partial CV sequences originated from greylag goose (*Anser anser*) and, based on the identity values, were amplified from GoCV genome, while others clustered with DuCV (*n* = 1, white stork) PiCV (*n* = 3, two from white storks and one from black-headed gull), TorCV1 (*n* = 4, three from great egret and one from spoonbill, *Platalea leucorodia*), SwCV (*n* = 1, mute swan) and GyoCV (*n* = 2, barn owl). The mentioned partial sequences represented high identity with corresponding references of the GenBank and/or the predicted *rep* of the here-determined complete genome sequences. DuCV sequences, obtained in this study, showed the highest identities with references originating from farmed ducks in China, South Korea and Thailand, while GoCV and PiCV sequences were closely related to those from Poland, as found by BLAST analyses. TorCV and SwCV have been described in wild birds in Hungary and Germany, respectively [11, 18]. Accession number of selected references chosen based on BLAST analysis are listed in Table 1.

One partial sequence, gained from mute swan, was distantly related (≤55.6% aa identity) with CVs, and was tentatively named swan-associated circovirus 1 (SwACV1). One of the two putative CyV sequences, originating from long-eared owl, showed high identity (94.6% nt, 97.2% aa) with bat faeces-associated CyV2 detected in Hungary (GenBank accession no. KY302865, Table 1). The other sequence, tentatively named stork-associated CyV1, was amplified from white stork and represented ≤73% aa identity with its closest related sequence, bat faeces-associated CyV2 from China (GenBank accession no. NC_038393, Table 1). Although both partial CyV sequences clustered with bat faeces-associated CyV2, these were only distantly related to each other (66.67% pairwise aa identity). Of note, the two bat faeces-associated CyV2 reference strains shared only 81.9% genome-wide identities. Partial Rep aa sequence of the other three, presumably unclassified CRESS DNA viruses (*Ciconia ciconia*-associated CRESS DNA virus 3 and *Ciconia ciconia*-associated CRESS DNA virus 4 from white storks and *Ardea cinerea*-associated CRESS DNA virus 1 from grey heron, *Ardea cinerea*) were distantly related to any reference sequences and were named also after the sampled bird (Table 1).

## Discussion

Agricultural intensification, high density of livestock and the decline of natural habitats has led to increased loads of relevant pathogens in the environment, water and soil [1, 2]. This can increase the likelihood of synanthropic birds shedding microbes, whether they become infected or serve as simple transmitters. The consequences of microbes entering the wild is unknown, but it certainly poses a threat that, owing to the overlap of habitats, may affect to farming or pet aviaries [1–3]. On the other hand, as is known, some pathogens endangering poultry and ornamental birds originate from wild animals, including highly pathogenic avian influenza virus [47].

The augmented agricultural activity favours synanthropic species, such as house sparrow (*Passer domesticus*), columbids, crows, starling (*Sturnus vulgaris*) or magpie (*Pica pica*), but more birds are forced to adopt to the built environment [48, 49]. In contrast to the ‘typical’ synanthropic animals, the sampled wild birds are less likely to come into close contact with industrial and backyard farming, but their behaviour depends on the populated geographical region and the availability of resources [50]. The sampled species white stork, black-headed gull or barn owl could also visit or live near settlements [50]. DuCV and PiCV was identified not only in these wild birds, but PiCV was also amplified from peregrine falcon. DuCV and GoCV have been previously found in wild birds of the respective avian groups (Anseriformes), including a project in which GoCV was detected in birds of variable lifestyle in Hungary [20, 51–54]. The white stork is taxonomically distant from the host species described carrying DuCV in the literature [54, 55]. PiCV were identified in columbids, except a raven suffering from lymphoid depletion and opportunistic infection, as well as in zoo birds, where close cohabitation and the constant presence of rock dove facilitate spillover [56–58].

In a previous study, the TorCV sequence was identified in little bittern sampled in Hungary, while TorCV sequences in the present project were amplified from great egret and spoonbill cloacal samples [18]. As in the case of any viruses characterized here, the infection of birds could not be proven and it is possible that the viruses only passed through the gastrointestinal tract of the birds without infection and replication. Nevertheless, considering that TorCV has only been found in freshwater birds so far, it is conceivable that this group of birds are natural hosts. SwCV has also been diagnosed in aquatic birds, in organ samples of swan [11]. A high incidence of SwCV was indicated at the site in Germany, but no further data were available and these are the first sequences since [11].

Genomic sequences of the two novel CVs, FulCV and GyoCV, originated from Strigiformes, and represent the closest relation with rodent-associated viruses. Based on the demarcation criteria of the International Committee on Taxonomy of Viruses, these viruses can be considered to belong to a new species, as both are relatively distant from the closest references from a genetic point of view [41]. However, dietary origin introduced by the prey cannot be ruled out, as in case of bat faeces-associated CyV2 found in a long-eared owl. Of note, the bat faeces-associated CyV2 sequence was also amplified from faeces of the associated mammal. Unfortunately, only partial sequences could be determined for two additional, potentially new members of the *Circoviridae* family that were found in mute swan and white stork specimens (swan-associated circovirus 1 and stork-associated CyV1), but these could be identified after complete genome sequence determination.

Although countless genome sequences are available, the knowledge of unclassified CRESS DNA viruses is incomplete [23, 24]. Thus, it is very difficult to assess the results. Six distantly related viruses were classified as CRESS DNA viruses from white stork, spoonbill and grey heron samples. This group of viruses has often been identified in environmental samples, including water and soil [6, 24]. Thus, water may also play an intermediary role in this case as well.

A relatively large proportion of the samples examined were of white stork origin (Additional file 1). This shows that white stork may be a transmitter of CVs, CyVs and unclassified CRESS DNA viruses. The breeding range of these long-distance migratory birds extends from Europe to northwestern Africa, and western and central Asia. The Hungarian Plain, along with Mediterranean areas with rivers and lakes are ideal stopover and breading habitats [59, 60]. Waterfowls prefer these watery areas rich in resources as well, but countless other migratory and resident birds reside in wetlands, which facilitate the transmission and shedding of pathogens, including CVs [61]. GoCV and PiCV sequences, obtained in the present study from greylag goose and white stork for example, represented the highest identities with sequences originating from Poland, suggesting that these viruses may have been transferred to variable geographical regions by migratory birds [61].

In conclusion, addressing the issues of the wildlife–agriculture interface is of great importance to maintain their balance and to approach the essence of the One Health concept. In parallel to the necessary expansion of agriculture, we must also consider the preservation of wildlife. Of course, it is not sufficient to focus on a single highly pathogenic microbe, but we must take numerous factors into account to protect poultry and wild bird populations. Birds living in the vicinity of poultry farms can serve as a link between the built environment and wilderness. Although CVs have mostly indirect, less dramatic effect on the host organism than highly pathogenic microbes, their presence cannot be ignored. Regular monitoring of both poultry and wild bird pathogens and hosts allows early recognition and effective intervention.

## Supplementary Information

**Additional file 1.** **The list of sampled wild birds and sites of sample collection.**

**Additional file 2.** **Sites of collection of avian samples in which circular, Rep-encoding single-stranded DNA virus sequences were identified.**

**Additional file 3.** **The list of the inverse primers used in this study. Letters F and R in the name of primers refer to the forward or reverse orientation of those.**

**Additional file 4.** **References used for read mapping and statistics of the next-generation sequencing.**

## Data Availability

All assembled sequence data generated or analysed during this study are available in the GenBank with accession numbers PV972686-PV972698 and PX097028-PX097048.
