# Supplementary material for: Occurrence of diverse circoviruses in wild birds in Hungary
Source: Vet Res. 2026 Jan 9;57:28. doi: 10.1186/s13567-025-01696-5 (PMC12879362; doi:10.1186/s13567-025-01696-5)
Supplement: Supplementary file 1 — Additional file 1. The list of sampled wild birds and sites of sample collection. [file 13567_2025_1696_MOESM1_ESM.docx]

**Additional file 1.** The list of sampled wild birds and sites of sample collection.

| **Sampled bird species** | **Number of samples** | **Site of collection** |
| --- | --- | --- |
| marsh tit (*Poecile palustris*) | 1 | Tömörd |
| Eurasian blackcap (*Sylvia atricapilla*) | 1 | Tömörd |
| marsh harrier (*Circus aeruginosus*) | 3 | Seregélyes |
| northern lapwing (*Vanellus vanellus*) | 1 | Sukoró |
| common Shelduck (*Tadorna tadorna*) | 3 | Seregélyes |
| mute swan (*Cygnus olor*) | 17 | Sumony, Siófok, Balatonszemes, Csorna, Gárdony, Pétfürdő, Somlóvásárhely |
| yellowhammer (*Emberiza citrinella*) | 1 | Tömörd |
| common chiffchaff (*Phylloscopus collybita*) | 2 | Tömörd |
| black-headed gull (*Chroicocephalus ridibundus*) | 46 | Gyál, Székesfehérvár |
| hooded crow (*Corvus cornix*) | 1 | Siófok |
| common buzzard (*Buteo buteo*) | 12 | Jánossomorja, Fehértó, Seregélyes, Pinnye, Lovasberény, Kunpeszér, Pécsely |
| song thrush (*Turdus philomelos*) | 1 | Tömörd |
| long-eared owl (*Asio otus*) | 29 | Jászberény, Pázmándfalu |
| white stork (*Ciconia ciconia*) | 78 | Szentendre, Tárnok, Érd, Pusztazámor, Egyházaskesző |
| common blackbird (*Turdus merula*) | 4 | Tömörd |
| Eurasian scops owl (*Otus scops*) | 1 | Káptalantóti |
| spotted redshank (*Tringa erythropus*) | 1 | Seregélyes |
| water rail (*Rallus aquaticus*) | 2 | Naszály |
| western barn owl (*Tyto alba*) | 16 | Jászberény, Hortobágy, Kisszőlős |
| corncrake (*Crex crex*) | 1 | Naszály |
| northern goshawk (*Accipiter gentilis*) | 4 | Kesztölc |
| common raven (*Corvus corax*) | 5 | Moha |
| common cuckoo (*Cuculus canorus*) | 1 | Seregélyes |
| Eurasian spoonbill (*Platalea leucorodia*) | 11 | Dinnyés |
| sparrowhawk (*Accipiter nisus*) | 1 | Sukoró |
| red-footed falcon (*Falco vespertinus*) | 1 | Seregélyes |
| little grebe (*Tachybaptus ruficollis*) | 1 | Seregélyes |
| little owl (*Athene noctua*) | 6 | Albertirsa, Jászberény, Budapest, Aba |
| tawny owl (*Strix aluco*) | 2 | Pázmándfalu, Nagybörzsöny |
| great egret (*Ardea alba*) | 42 | Dunavarsány, Dinnyés |
| greylag goose (*Anser anser*) | 107 | Agárd, Gárdony, Sukoró |
| Eurasian wren (*Troglodytes troglodytes*) | 2 | Tömörd |
| wood sandpiper (*Tringa glareola*) | 2 | Gárdony/ Dinnyés |
| short-eared owl (*Asio flammeus*) | 49 | Jászberény, Hortobágy |
| white-tailed eagle (*Haliaeetus albicilla*) | 1 | Sárbogárd |
| goldcrest (*Regulus regulus*) | 3 | Tömörd |
| common snipe (*Gallinago gallinago*) | 1 | Gárdony/ Dinnyés |
| common starling (*Sturnus vulgaris*) | 2 | Sarród/ Fertőújlak, Kópháza |
| mediterranean gull (*Ichthyaetus melanocephalus*) | 9 | Gyál |
| grey heron (*Ardea cinerea*) | 11 | Dunavarsány, Várpalota |
| mallard (*Anas platyrhynchos*) | 10 | Debrecen, Szeged, Budapest, Mezőberény |
| little bittern (*Botaurus minutus*) | 1 | Naszály |
| peregrine falcon (*Falco peregrinus*) | 3 | Lábatlan, Csókakó |
| rook (*Corvus frugilegus*) | 5 | Nagycenk, Pázmándfalu, Székesfehérvár |
| common kestrel (*Falco tinnunculus*) | 64 | Albertirsa, Jászberény, Seregélyes, Pázmándfalu, Székesfehérvár, Gárdony, Sárkeresztúr, Aba, Bánd, Szentkirályszabadja, Ecser |
| European robin (*Erithacus rubecula*) | 5 | Tömörd |
| red-tailed hawk (*Buteo jamaicensis*) | 1 | Gödöllő |
| no data available | 17 | no data available |
