# Supplementary material for: Occurrence of diverse circoviruses in wild birds in Hungary
Source: Vet Res. 2026 Jan 9;57:28. doi: 10.1186/s13567-025-01696-5 (PMC12879362; doi:10.1186/s13567-025-01696-5)

**Additional file 2.** Sites of collection of avian samples in which circular, Rep-encoding single-stranded DNA virus sequences were identified.

| **Virus name** | **Virus strain** | **GenBank acc. no.** | **Sampled bird** | **Site of collection** |
| --- | --- | --- | --- | --- |
| ***duck circovirus*** | ***DuCV-Hun1*** | ***PV972690*** | ***white stork*** | ***Érd*** |
| duck circovirus | DuCV-Hun2 | PX097028 | white stork | Érd |
| ***pigeon circovirus*** | ***PiCV-Hun1, n=2*** | ***PV972689*** | ***peregrine falcon*** | ***Lábatlan*** |
| pigeon circovirus | PiCV-Hun2 | PX097029 | white stork | Pusztazámor |
| pigeon circovirus | PiCV-Hun3 | PX097030 | white stork | Pusztazámor |
| pigeon circovirus | PiCV-Hun4 | PX097031 | black-headed gull | Székesfehérvár |
| goose circovirus | GoCV-Hun4 | PX097032 | greylag goose | Sukoró |
| goose circovirus | GoCV-Hun5 | PX097033 | greylag goose | Sukoró |
| goose circovirus | GoCV-Hun6 | PX097034 | greylag goose | Dinnyés |
| goose circovirus | GoCV-Hun7 | PX097035 | greylag goose | Sukoró |
| ***swan circovirus*** | ***SwCV-Hun1, n=6*** | ***PV972694*** | ***mute swan*** | ***Csorna*** |
| swan circovirus | SwCV-Hun2 | PX097036 | mute swan | Csorna |
| ***gull circovirus*** | ***GuCV-Hun1*** | ***PV972695*** | ***black-headed gull*** | ***Gyál*** |
| ***little bittern circovirus*** | ***TorCV1-Hun2*** | ***PV972691*** | ***great egret*** | ***Dinnyés*** |
| ***little bittern circovirus*** | ***TorCV1-Hun3*** | ***PV972692*** | ***great egret*** | ***Dinnyés*** |
| ***little bittern circovirus*** | ***TorCV1-Hun4*** | ***PV972693*** | ***great egret*** | ***Dinnyés*** |
| little bittern circovirus | TorCV1-Hun5 | PX097037 | great egret | Dinnyés |
| little bittern circovirus | TorCV1-Hun6 | PX097038 | great egret | Dinnyés |
| little bittern circovirus | TorCV1-Hun7 | PX097039 | great egret | Dinnyés |
| little bittern circovirus | TorCV1-Hun8 | PX097040 | spoonbill | Dinnyés |
| ***long-eared owl-associated circovirus 1*** | ***le-owlCV1-Hun1*** | ***PV972686*** | ***long-eared owl*** | ***Jászberény*** |
| ***barn owl-associated circovirus 1*** | ***barn-owlCV1-Hun1*** | ***PV972687*** | ***barn owl*** | ***Hortobágy*** |
| ***barn owl-associated circovirus 1*** | ***barn-owlCV1-Hun2*** | ***PV972688*** | ***barn owl*** | ***Kisszőlős*** |
| barn owl-associated circovirus 1 | barn-owlCV1-Hun3 | PX097041 | barn owl | Hortobágy |
| barn owl-associated circovirus 1 | barn-owlCV1-Hun4 | PX097042 | barn owl | Kisszőlős |
| swan-associated circovirus 1 | SwACV1-Hun1 | PX097043 | mute swan | Somlóvásárhely |
| stork-associated cyclovirus 1 | StorkACyV1-Hun1 | PX097044 | white stork | Pusztazámor |
| bat faeces associated cyclovirus 2 | BatACyV2-Hun1 | PX097045 | long-eared owl | Pázmándfalu |
| ***Ciconia ciconia-associated CRESS DNA virus 1*** | ***Cic-cic-CRESS1-Hun1*** | ***PV972697*** | ***white stork*** | ***Pusztazámor*** |
| ***Ciconia ciconia-associated CRESS DNA virus 2*** | ***Cic-cic-CRESS2-Hun1*** | ***PV972698*** | ***white stork*** | ***Tárnok*** |
| Ciconia ciconia-associated CRESS DNA virus 3 | Cic-cic-CRESS3-Hun1 | PX097046 | white stork | Pusztazámor |
| Ciconia ciconia-associated CRESS DNA virus 4 | Cic-cic-CRESS4-Hun1 | PX097047 | white stork | Pusztazámor |
| ***Platalea leucorodia-associated CRESS DNA virus*** | ***Plat-leu-CRESS-Hun1*** | ***PV972696*** | ***spoonbill*** | ***Dinnyés*** |
| Ardea cinerea-associated CRESS DNA virus 1 | Ar-Ci-CRESS1-Hun1 | PX097048 | grey heron | Dunavarsány |


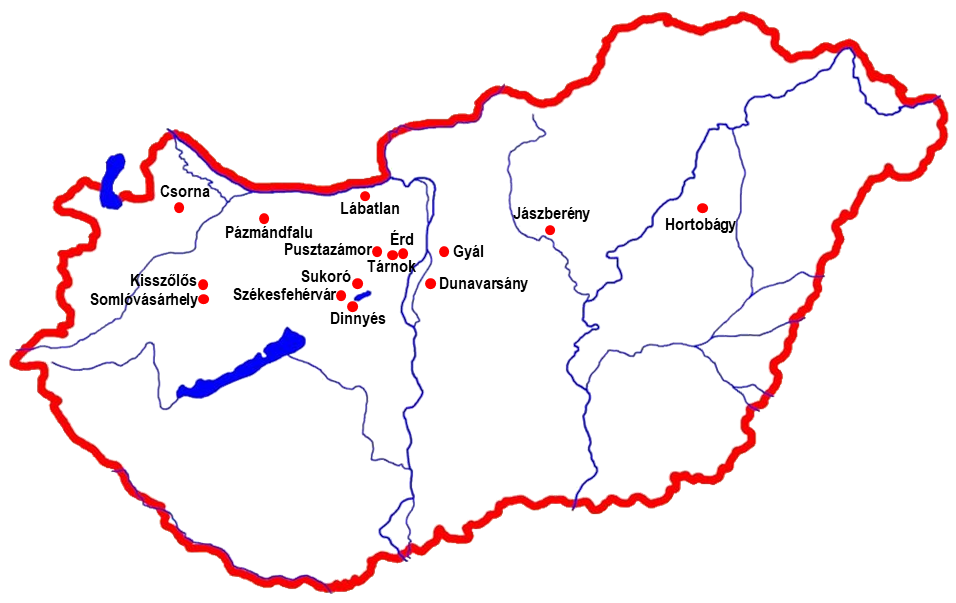

Supplement: Supplementary file 2 — Additional file 2. Sites of collection of avian samples in which circular, Rep-encoding single-stranded DNA virus sequences were identified. [file 13567_2025_1696_MOESM2_ESM.docx]
