# Supplementary material for: Occurrence of diverse circoviruses in wild birds in Hungary
Source: Vet Res. 2026 Jan 9;57:28. doi: 10.1186/s13567-025-01696-5 (PMC12879362; doi:10.1186/s13567-025-01696-5)
Supplement: Supplementary file 3 — Additional file 3. The list of the inverse primers used in this study. Letters F and R in the name of primers refer to the forward or reverse orientation of those. [file 13567_2025_1696_MOESM3_ESM.docx]

**Additional file 3.** The list of inverse primers used in this study. Letters F and R in the name of primers refer to the forward or reverse orientation of those.

| **Virus name** | **Primer name** | **Primer sequence (5'-3')** | **Primer annealing** |
| --- | --- | --- | --- |
| duck circovirus | DuCV-bbF1-1 | GAGTCGACATACCTTCGAGTTGG | 57 °C |
|  | DuCV-bbF1-2 | GAGTCGACATACCTGAGAGTTGG |  |
|  | DuCV-bbR1 | AGAGAGCCAGGCTCTTCCTC |  |
| pigeon circovirus | PiCV-bbF1 | CGGAAGTCATCGTSATCACGGG | 58 °C |
|  | PiCV-bbF2 | TACGTCAARTATGGGCGTGG |  |
|  | PiCV-bbR1 | TCGCTCCGGTTTCCCTTCG |  |
|  | PiCV-bbR2 | CATTGCTCTTCCGGCTTTCAC |  |
| goose circovirus | GoCV-bbF1 | GAGCTCGGGGATCTGACGAAG | 59 °C |
|  | GoCV-bbF1 | AGACAGCCAGGCTCTTCCTC |  |
| swan circovirus | SwCV-bbF1 | AGGAAGAGCATGGCTGTCTCG | 59 °C |
|  | SwCV-bbF2 | ATCTGAACGCCGCTGCATCC |  |
|  | SwCV-bbR1 | CCCAGGTTCTCTTCAAGGGC |  |
|  | SwCV-bbR2 | TTCTCTTCGTCAGATCCCACAGC |  |
| gull circovirus | GullCV-bbF1 | AAATGAGTGAAATCGCGCGAG | 56 °C |
|  | GullCV-bbF2 | AGTGAAGTCTACGTCAAGTATGGG |  |
|  | GullCV-bbR1 | CTCGGAGTACCGATCATGGTAAG |  |
| little bittern circovirus | littlebitternCV-bbF2 | GCTCATCACTTATGGAGGTCGC | 57 °C |
|  | littlebitternCV-bbR2 | GCTTGAAGCATTGACACAGCATC |  |
| long-eared owl-associated circovirus 1 | BFDCV-like-bbF1 | GGCTCGACACCACGTGACTTC | 60 °C |
|  | BFDCV-like-bbF2 | AGTTCGACAGCGCTGTTGCC |  |
|  | BFDCV-like-bbR1 | TTCTTCAGCGCGGTCAGACG |  |
|  | BFDCV-like-bbR2 | TCTCGAAGTGCGCTCGCTTC |  |
| barn owl-associated circovirus 1 | RoCV-bbF2 | CAGAGCCTGCCAGATACTCCG | 60 °C |
|  | RoCV-bbR2 | CAGCGAGGTGCTCTTCCCC |  |
| swan-associated circovirus 1 | DuCV-like-bbF1 | TGCCCGGAAGTTCCCAGTGC | 63 °C |
|  | DuCV-like-bbR1 | TCGAGCATAGCCGCGCACTC |  |
|  | DuCV-like-bbF2 | TCAGCTGATGTGCGGCACGC |  |
|  | DuCV-like-bbR2 | CTTCAGGTCGTTCCTCTGGCCC |  |
| stork-associated cyclovirus 1 | CyV1-bbF1 | ACTCATTGGCGCCTTACATCCTG | 60 °C |
|  | CyV1-bbR1 | GATTGTCTGCGTCTGTCCCGT |  |
|  | CyV1-bbF2 | AGAGTATCGCAGAGCAATACCCTGG |  |
| bat faeces associated cyclovirus 2 | BatCV-bbF2 | AGAAGCCAGACAGATCGATCCCG | 60 °C |
|  | BatCV-bbR2 | TTGTCCTTGTACCACAGGGCTTC |  |
| Ciconia ciconia-associated CRESS DNA virus 1 | CRESS2-bbR2 | TACGATTGGATTCCACGGGC | 57 °C |
|  | CRESS2-bbF1 | AGATACTGGTGTTGGCAAGTCC |  |
|  | CRESS2-bbR1 | ACAGTCGTCTTCCAAGATCGC |  |
| Ciconia ciconia-associated CRESS DNA virus 2 | CRESS1-bbF1 | GTCGAAGAGGTGACTTGGACGC | 60 °C |
|  | CRESS1-bbR1 | CTTGAGCGGGTCGAGTACCG |  |
| Ciconia ciconia-associated CRESS DNA virus 3 | CyV2-bbF1 | AAGATACTCTACGGAGATCCAGG | 58 °C |
|  | CyV2-bbR1 | AACCTCTGTCTTCCATCCTCTTC |  |
|  | CyV2-bbR2 | TGATCTTCTGCAACTCTCTTGAGG |  |
| Ciconia ciconia-associated CRESS DNA virus 4 | CRESS3-bbF2 | TGAAGCAACGTACGAAGCGGC | 67 °C |
|  | CRESS3-bbF1 | TAGCGAGAGCATGTGATGCAGC |  |
|  | CRESS3-bbR1 | TCCCAATGTGCTGCCTCATGTAG |  |
| Platalea leucorodia-associated CRESS DNA virus | CRESS6-bbF1 | TTGCTGAGGAACAACCTGCTG | 57 °C |
|  | CRESS6-bbR1 | CCACACACGTAAGTCTCATCGTC |  |
|  | CRESS6-bbF2 | CAAAGCATGGCTGGATGTAACTG |  |
|  | CRESS6-bbR2 | TTCATCACTCCTTGCTTGGACC |  |
| Ardea cinerea-associated CRESS DNA virus 1 | CRESS4-bbF1 | ATTGATCGAGGCTGCACCTG | 58 °C |
|  | CRESS4-bbR1 | TTGAGATCGTTTCGTGTTCCTGC |  |
|  | CRESS4-bbF2 | AGATAGTCGAGCCCATCGCTC |  |
|  | CRESS4-bbR2 | TGTAATGTGCCGTGATCATGCC |  |
