# Supplementary material for: Occurrence of diverse circoviruses in wild birds in Hungary
Source: Vet Res. 2026 Jan 9;57:28. doi: 10.1186/s13567-025-01696-5 (PMC12879362; doi:10.1186/s13567-025-01696-5)
Supplement: Supplementary file 4 — Additional file 4. References used for read mapping and statistics of the next-generation sequencing. [file 13567_2025_1696_MOESM4_ESM.docx]

**Additional file 4.** References used for read mapping and statistics of the next-generation sequencing.

| **Virus name** | **Assembly type** | **Total read number** | **Assembled read number** | **Mean coverage** |
| --- | --- | --- | --- | --- |
| **long-eared owl-associated circovirus 1, PV972686** | *de novo* assembled | 286,822 | 281,404 | 10,744 |
| **barn owl-associated circovirus 1, PV972687** | *de novo* assembled | 416,842 | 2,075 | 123 |
| **barn owl-associated circovirus 1, PV972688** | *de novo* assembled | 198,274 | 148,535 | 5,063 |
| **pigeon circovirus,**  **PV972689, sample 10** | *de novo* assembled | 379,552 | 297,372 | 17,277 |
| **pigeon circovirus,**  **PV972689, sample 11** | *de novo* assembled | 433,064 | 320,866 | 15,631 |
| **duck circovirus, PV972690** | *de novo* assembled | 270,306 | 62,207 | 2,991 |
| **little bittern circovirus, PV972691** | map to reference MZ710934 | 204,942 | 198,346 | 7,777 |
| **little bittern circovirus, PV972692** | map to reference MZ710934 | 169,820 | 134,573 | 5,571 |
| **little bittern circovirus, PV972693** | map to reference MZ710934 | 275,774 | 265,012 | 11,697 |
| **swan circovirus, PV972694, sample 7E** | map to reference EU056309 | 259,652 | 45,635 | 2,601 |
| **swan circovirus, PV972694, sample 7G** | map to reference EU056309 | 390,374 | 380,066 | 19,552 |
| **swan circovirus, PV972694, sample 7H** | map to reference EU056309 | 346,970 | 89,481 | 5,159 |
| **swan circovirus, PV972694, sample 8A** | map to reference EU056309 | 401,204 | 391,536 | 21,071 |
| **swan circovirus, PV972694, sample 8C** | map to reference EU056309 | 312,538 | 242,363 | 14,085 |
| **swan circovirus, PV972694, sample 8E** | map to reference EU056309 | 361,752 | 331,430 | 18,302 |
| **gull circovirus, PV972695** | *de novo* assembled | 66,910 | 40,532 | 1470 |
| **Ciconia ciconia-associated CRESS DNA virus 1, PV972697** | *de novo* assembled | 117,170 | 114,792 | 4,057 |
| **Ciconia ciconia-associated CRESS DNA virus 2, PV972698** | *de novo* assembled | 291,214 | 74,311 | 3,688 |
| **Platalea leucorodia-associated CRESS DNA virus, PV972696** | *de novo* assembled | 35,964 | 34,120 | 1370 |
